# Relative Effects of Juvenile and Adult Environmental Factors on Mate Attraction and Recognition in the Cricket, *Allonemobius socius*

**DOI:** 10.1673/031.010.9001

**Published:** 2010-07-04

**Authors:** Alexander E. Olvido, Pearl R. Fernandes, Timothy A. Mousseau

**Affiliations:** ^1^ Division of Science, Gainesville State College (Oconee campus), 1201 Bishop Farms Parkway, Watkinsville, Georgia, 30677, USA; ^2^ Division of Science, Mathematics and Engineering, University of South Carolina Sumter, Sumter, South Carolina, 29150, USA; ^3^ Department of Biological Sciences, University of South Carolina — Columbia, Columbia, South Carolina, 29208, USA

**Keywords:** heritability, maternal effect, mating call, Orthoptera, phonotaxis, reaction norm

## Abstract

Finding a mate is a fundamental aspect of sexual reproduction. To this end, specific-mate recognition systems (SMRS) have evolved that facilitate copulation between producers of the mating signal and their opposite-sex responders. Environmental variation, however, may compromise the efficiency with which SMRS operate. In this study, the degree to which seasonal climate experienced during juvenile and adult life-cycle stages affects the SMRS of a cricket, *Allonemobius socius* (Scudder) (Orthoptera: Gryllidae) was assessed. Results from two-choice behavioral trials suggest that adult ambient temperature, along with population and family origins, mediate variation in male mating call, and to a lesser extent directional response of females for those calls. Restricted maximum-likelihood estimates of heritability for male mating call components and for female response to mating call appeared statistically nonsignificant. However, appreciable “maternal genetic effects” suggest that maternal egg provisioning and other indirect maternal determinants of the embryonic environment significantly contributed to variation in male mating call and female response to mating calls. Thus, environmental factors can generate substantial variation in *A. socius* mating call, and, more importantly, their marginal effect on female responses to either fast-chirp or long-chirp mating calls suggest negative fitness consequences to males producing alternative types of calls. Future studies of sexual selection and SMRS evolution, particularly those focused on hybrid zone dynamics, should take explicit account of the loose concordance between signal producers and responders suggested by the current findings.

## Introduction

A well-supported idea for mate discrimination is avoidance of unfit hybrids. In birds, for example, genetically based courtship display traits may have evolved to prevent heterospecific mating that likely yields hybrid offspring with lower-than-average fitness (Dobzhansky 1937). While appearing to explain many contemporary patterns of reproductive isolation, Dobzhansky's theory of isolating mechanisms sheds little light on sexual selection and its possible role in speciation, especially in the common case where related species overlap in their geographic distributions (Andersson 1994).

In contrast, the specific-mate recognition concept puts forth the notion that secondary sexual traits evolved to promote the pairing of compatible genotypes, e.g. coordination of mating signal and intraspecific responders (Paterson 1985). Hence, behaviors such as courtship displays can signal sexual readiness to genetically compatible individuals in a population, with the probable result that such matings produce high-fitness offspring. Unlike the avoidance-of-unfit-hybrids explanation, specific-mate recognition provides a conceptually straight-forward framework for studying sexual selection within a species and initiation of the speciation process (Andersson 1994).

The southern ground cricket, *Allonemobius socius* (Orthoptera: Gryllidae), presents opportunities for investigating the evolution of a specific-mate recognition system (SMRS). A small (less than 2 cm in anterior-posterior length) terrestrial insect, *A. socius* inhabits fields and woodlands throughout the southeastern region of North America (Howard and Furth 1986).

Allozyme studies indicated *A. socius* forms part of a complex of two sister species meeting in a hybrid zone from southern New Jersey through Illinois (∼40° latitude) (Howard and Furth 1986; Howard and Waring 1991). While genital morphology and mating behavior prove useful in characterizing other insects (Walker 1957; Lloyd 1984; Bonduriansky 2001), no such clear distinctions exist between *A. socius* and its more northern congener, *A. fasciatus* (Howard and Furth 1986; Veech et al. 1996). Currently, the only quick and reliable method to distinguish the two species in the wild is collecting by geographic site (Howard and Furth 1986; DJ Howard New Mexico State University (Las Cruces, NM), personal communication).

Given its widespread North American distribution, *A. socius* varies substantially in development rate, morphology, and reproductive behavior (Mousseau and Roff 1989, 1995). Northern A *socius* populations produce one generation per year, while more southern populations produce two or more (Howard and Furth 1986; Walker and Masaki 1989; Mousseau and Roff 1989; Mousseau 1991). Like in other gryllids, *A. socius* males stridulate, or rub their forewings together, to emit a chirp-like mating call that functions as a signal to nearby females of the male's readiness to mate. While the immediate effects of ambient temperature on insect mating calls are well-known, i.e. since Brooks (1882) and Dolbear (1897), only recently have researchers begun to explore how environmental variation during the juvenile stages of the life cycle might shape reproductive behavioral reaction norms, e.g. degree to which male mating call varies systematically with ambient temperature (Whitesell and Walker 1978; Olvido and Mousseau 1995; Grace and Shaw 2004).

Environmental variation might also affect female responses to male mating call. Olvido and Wagner (2004) showed that *A. socius* females preferred experimentally manipulated long-chirp mating calls, i.e. those with above-average chirp duration, and paid surprisingly little attention to variation in chirp rate, i.e. the number of chirps per seconds in a mating call. Since chirp duration and other components of *A. socius* male mating call vary with temperature and rearing environment (Olvido and Mousseau 1995), it is quite possible that female preferences also vary with ambient temperature and/or rearing environment.

In this study, the effects of genetic and non-genetic factors on the SMRS of *A. socius* were examined as a continuing effort to assess, ultimately, their contributions to this species' persistent hybridization with *A. fasciatus.* the hypothesis of environmentally mediated phenotypic and genetic coupling between male mating call and relative preference of females for mating call was tested by, first, rearing split broods of *A. socius* juveniles in different laboratory “seasonal” environments and, later, analyzing their sex-specific behaviors across different ambient temperatures. If, indeed, the current *A. fasciatus-A. socius* hybrid zone is maintained primarily by *A. socius* females migrating northward into *A. fasciatus* populations and mating preferentially with *A. fasciatus* males, then a weakly evolved SMRS would be expected in *A. socius,* i.e. weak or absent phenotypic and/or genetic coupling between *A. socius* mating call and female relative preference for mating call, all else being equal. However, if the *A. fasciatus-A. socius* hybrid zone persists mainly for reasons other than promiscuous *A. socius* females mating with heterospecifics, then significant phenotypic and/or genetic coupling between *A. socius* mating call and female relative preference for mating call would be expected.

## Methods

### Field collection and animal husbandry

Cricket stocks were derived from individuals collected from two sites located approximately 170 km apart (in Columbia and Travelers' Rest, South Carolina, USA). Gravid field-caught females were housed singly and allowed to oviposit *ad libitum* in cheesecloth. Field-caught juveniles were reared in several small-group cages and maintained in the laboratory at 31° C with a 15-hr daily light cycle, or DLC. From each maternal line (established either from field-caught gravid females or from singly paired males and females that had matured under laboratory conditions), the following generation was reared exclusively at 31° C and a 15-hr DLC to minimize any confounding genotype-by-environment interactions in subsequent phenotypic analyses.

For the second laboratory-reared generation (i.e. two generations removed from the field), one-half of a brood of newly hatched nymphs was reared exclusively under spring-like conditions (24° C, 11-hr DLC) in several small-group cages and, simultaneously, the other half exclusively under summer-like conditions (31° C, 15-hr DLC) in several small-group cages. As in previous generations, females were separated from males before the penultimate juvenile stage to assure virginity of subjects in the ensuing behavioral assays. Adult crickets were maintained at temperatures and photoperiods of their respective “juvenile” environments through all experimental trials.

### Measuring cricket sexual behaviors

Each male cricket was allowed 5–20 minutes to acclimate to a particular ambient temperature before its continuously produced mating call was recorded at that temperature in an echo-dampened chamber. In total, 424 males (1–5 weeks from adult emergence) were recorded calling at 24°, 28°, and 31° C. A haphazardly chosen 10-second sample of each mating call was later imported to a desktop computer as an 8-bit, 22-kHz WAV file and analyzed with “Spectrogram 2.2” (©1994, RS Home) and “Wave for Windows 2.03” (©1993, Turtle Beach Systems, www.turtlebeach.com) graphical analysis software. The four mating call components of interest were chirp rate (*crat*, number of chirps per second), pulses per chirp (*ppc*, number of acoustic pulses per chirp), chirp duration (*cdur*, number of seconds from beginning to end of each chirp), and dominant frequency (*dfrq*, frequency of oscillation at peak power spectral density).

Which mating call to use in phonotaxis trials was determined beforehand via principal components analysis (i.e. PRINCOMP procedure in SAS/STAT) of the four mating call components mentioned above. For each population, the two unmanipulated mating calls having their first principal component (= PC1, which explained 54% of total variation in mating call) most closely match the mean PC1 of males calling at 24° C and at 31° C (“cold” and “hot” songs, respectively) were chosen. Analysis of eigenvectors indicated that *crat* and *cdur* both loaded equally well on PC1, but from opposite directions (-0.505 for *crat* and 0.508 for *cdur*), whereas eigenvectors for *ppc* and *dfrq* were lower (0.382 and -0.386, respectively). Thus, the chosen stimuli seemed to differ mainly in chirp rate and chirp duration, which were also strongly correlated with each other (r = -0.4989).

Each singly tested female cricket (1–5 weeks after adult emergence) experienced simultaneous playback of “hot” and “cold” call stimuli (standardized to 70 db SPL at approximately 1 m from each speaker) through two three-minute trials — once at 24° C and, several days later, again at 31° C (after a 5- to 20-minute acclimation period for each trial). For each test subject at each ambient temperature, left-right orientation of speakers was randomized in the square (1.44-m^2^ floor area) anechoic observation chamber such that a left-corner speaker would broadcast a “hot” mating call in one trial before being switched in a subsequent trial with the other speaker broadcasting a “cold” mating call at the adjacent corner. Given the initially large sample size (> 300 females) and labor-intensive nature of measuring phonotaxis, 3–7 days were allowed between repeated observations. Positive phonotaxis was scored when the test subject approached within 30 cm of a speaker. The freely accessible space between speakers (approximately 45 cm in width) assured that female phonotaxis was measured independently of either call stimulus. Through 1086 playback trials videotaped under low-intensity red light (to minimize any confounding visual cues), 300 females yielded score-able phonotaxis.

Female response to call stimuli was characterized in two different ways. First, phonotaxis was quantified as the inverse of time elapsed (in seconds) for a female test subject to approach its first speaker, i.e. initial choice (*init*): Positive initial-choice scores indicated attraction to the speaker broadcasting (exclusively) a “hot” mating call, while negative scores indicated attraction to one broadcasting (exclusively) a “cold” mating call, regardless of relative orientation of speakers. Initial choice, thus, appeared to measure a female *A. socius*'s general degree of attentiveness to “hot” and “cold” variations of intraspecific mating call.

Alternatively, female phonotaxis was quantified as the difference in time (in seconds) a female test subject loitered between the two speakers playing their respective call stimuli simultaneously: Positive loitering behavior scores indicated greater time spent within 30 cm of the speaker broadcasting exclusively the “hot” mating call, and negative scores indicated greater time spent within 30 cm of the speaker broadcasting exclusively the “cold” mating call, regardless of relative orientation of speakers. Thus, loitering behavior (*lngr*) appeared to measure relative strength of directional preference of females for either the “hot” or “cold” mating call stimulus.

As a matter of clarification, the measurement of relative preferences presumed that, given the audible and quantifiable differences in “hot” versus “cold” call stimuli, our female test subjects would prefer one or the other call stimulus type. The main issue was assessing how malleable a female's relative preference might be, as relative preference may reflect a female's absolute preference for male calls of a specific chirp rate, chirp duration, etc. Assessing the malleability of female absolute preferences or the relative importance for individual mating call components will require a far greater investment of resources than was possible in the current work.

To discount a possible left- versus right-side walking bias in *A. socius* females, each test subject was allowed to wander in the observation chamber without playback of call stimuli. For each test subject, silent trials were conducted in random order with respect to phonotaxis trials and also under red-light conditions, but at room temperature (25–27° C). The magnitude of walking side bias was quantified as a proportion of the total time within a three-minute observation period that a test subject loitered within 30 cm of either silent speaker [(t_LEFT_ - t_RIGHT_)/(t_LEFT_ + t_RIGHT_)]. Positive scores indicated for left-side wandering bias, and negative scores indicated right-side wandering bias. Females failing to approach either silent speaker within the alloted three minutes were excluded from further analysis (sample size in silent trials, *n*_TRIAL0_, was 114 females).

### Phenotypic analyses of adult behaviors

To minimize bias from genotype-by-environment interactions, all phenotypic analyses of reproductive behavior was limited to sexually mature crickets from the second laboratory-reared generation. Borrowing a technique from a van der Waerden normal scores analysis (Conover 1999), all raw variates were transformed to their normalized ranks before performing analysis-of-variance, or ANOVA, testing (see also Olvido and Wagner 2004). Hence, the phenotypic analyses explicitly satisfy two fundamental assumptions of ANOVA models, namely that all treatments for a given factor share a common mean (here, overall µ = 0) and have comparable variance (i.e. overall s^2^ approximates unity).

To facilitate analysis of female relative preference for mating call, and as dictated by the repeated-measures design, a cross-nested ANOVA was created with one repeated factor:

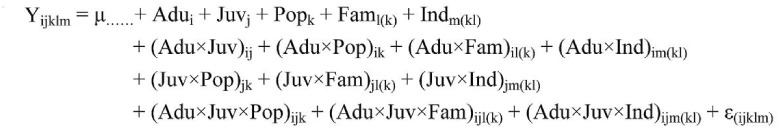

where Y_ijklm_ indicates the female trait of interest, Adu_i_ indicates adult ambient temperature (repeated factor; fixed effect), Juv_j_ indicates rearing environment (fixed effect), Pop_k_ indicates population origin (random effect), Fam_l(k)_ indicates within-population family origin or maternal line (random effect), and Ind_m(kl)_ indicates the individual test subject nested within population and family (random effect), and µ..... and ε_(ijklm)_ indicate the common mean and error term, respectively. Having had recorded female behaviors only once at each ambient temperature, an ANOVA model was constructed that lacked an independent error term. Interaction terms were considered random when involving at least one random main effect. Since repeated-measures ANOVA requires that each test subject complete both phonotaxis-temperature trials, data from only 160 females (× 2 ambient temperatures = 320 repeated observations) of the total 300 test subjects were analyzed.

Female phonotaxis was analyzed without age as a covariate because an earlier study showed consistency in call stimulus preference through 90% of the adult life of *A. socius* females: Three-week old females preferring long-chirp calls still preferred those same calls at 17 weeks of age, with only a marginal decline in time spent near speakers broadcasting those preferred calls (Olvido and Wagner 2004).

The same cross-nested, repeated-measures ANOVA was applied to the analysis of male mating calls. As required of repeated-measures designs, only data from males completing all temperature treatments (N = 266 males × 3 ambient temperatures = 798 observations) were analyzed.

The GLM procedure was used in SAS/STAT to obtain information on ANOVA degrees of freedom and Type III mean squares, from which observed *F*-values were calculated. (Tables 3 and 4 contain explicit description of each *F* test.) For each observed *F*-value through the distribution functions, p-values were obtained in Stat-SAK 2.14 (©1986, GE Dallal).

### Pedigree Analysis

A series of Fortran-77 programs, known collectively as “Multiple Trait Derivative-Free Restricted Maximum Likelihood,” or simply “MTDFREML” (Boldman et al. 1995), were run to evaluate mixed-model equations that partition phenotypic variance specifically into additive-genetic and other model variance components. The particular mixed-model equations model applied is known as the full animal model with no covariates for inbreeding of offspring with dams (most similar to Model 4 in Ferreira et al. (1999)), and can be expressed as:

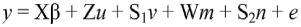

where *y* represents a vector of observations (for a single trait), β is a vector of fixed effects (which include population origin, adult ambient temperature, and juvenile rearing environment), *u* indicates a vector of random animal (direct) effects, *v* indicates a vector of random animal permanent environmental effects, *m* indicates a vector of random maternal (indirect) genetic effects, *n* indicates a vector of random maternal permanent environmental effects, *e* indicates a vector of random residual effects, and X, Z, S_1_, W, and S_2_ represent association matrices for fixed, random direct, animal permanent environmental, random indirect, and maternal permanent environmental effects, respectively. (An association matrix ties performance data to particular test subjects and treatment groups, as well as specifies familial relationships.)

To document how juvenile rearing environment might affect estimates of heritability and other proportional variances, separate MTDFREML analyses were performed on spring- and summer-reared crickets (even though both rearing groups shared the same pedigree). The pedigree was composed of 1,362 individuals (including grandsires, granddams, sires, and dams) and a total of 752 individuals from the second laboratory-reared generation (446 males and 300 females recorded multiple times) in the cumulative performance data set that was sub-sampled to obtain separate quantitative-genetic parameter estimates from spring-versus summer-reared groups. Each MTDFREML session stopped when variance of the simplex algorithm, Var (21ogΛ), reached 1 × 10^-6^. We presumed convergence at a global maximum when both the simplex values and heritability point estimates from consecutive MTDFREML sessions remained unchanged at the second decimal place (LD Van Vleck, personal communication, USDA-ARS, University of Nebraska at Lincoln). For each proportional variance, the 95% confidence interval was approximated as twice the REML standard error of the point estimate, and determined statistical significance when that interval excluded zero. Ferreira et al. (1999) and Boldman et al. (1995) provide more explicit descriptions of MTDFREML programs and their correct implementation. The pedigree and phenotypic data file is available in Appendix 1 online, or upon request to the first author.

**Figure 1.**
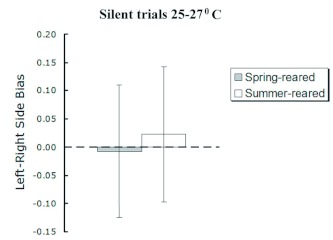
Absence of left- versus right-side walking bias of *Allonemobius socius* females during silent trials (mean ± 1 SE, in seconds). A positive score indicates that a female spent a greater proportion of the three-minute trial period wandering near the right-side corner of the observation chamber (*n*_TRIAL0_ = 114 females). See Methods for full description of measurement protocols. High quality figures are available online

## Results

Visual analysis of results from the silenttrials indicated no left- or right-side walking bias in female *A. socius* used in this study. Regardless of rearing environment, *A. socius* females were as likely to wander near the left corner of the observation chamber as the right corner: walking scores for both spring- and summer-reared females hovered near zero (Figure 1).

**Figure 2.**
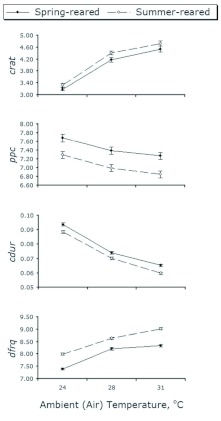
Environmental effects on *Allonemobius socius* male mating call (mean ± 1 SE). Full-sibling males were reared in paired treatments as juveniles exclusively under “spring” versus “summer” conditions. Each male's mating call was recorded only once in each of three adult ambient temperatures (*n* = 266 males). Traits codes are *crat*: chirp rate (i.e. number of chirps per second) of male mating call; *ppc*: number of acoustic pulses per chirp of male mating call; *cdur*: chirp duration in seconds of male mating call; *dfrq*: dominant frequency in kilohertz of male mating call. See Methods for full description of trait codes and measurement protocols. High quality figures are available online.

### Factors affecting male mating call

Adult ambient temperature clearly affected all four components of male mating call. Chirp rate and dominant frequency of mating call tended to increase with increasing ambient temperature (Figure 2), though two- and three-way interactions with population origin, rearing environment, family origin, and individual suggest non-linear ambient temperature effects on these two male traits (*crat* and *dfrq* in Table 1, construction of F-test is given in Table 3). On the other hand, chirp duration and number of pulses per chirp significantly varied only with adult temperature (Adu on *cdur* and *ppc* in Table 1), indicating that chirp duration and pulses-per-chirp tend to decline uniformly as ambient temperature increases (Figure 2).

Compared with adult ambient temperature, juvenile rearing environment had considerably less effect on male mating call. Only pulses per chirp registered significant variation due to juvenile rearing environment (Juv on *ppc* in Table 1, construction of F-test in Table 3). The two-way interactions of juvenile rearing environment with adult ambient temperature and with population origin (Adu × Juv and Juv × Pop) appeared statistically non-significant, which supports the consistently higher number of pulses per chirp in spring- versus summer-reared males (Figure 2). Moreover, the significant two-way interaction of juvenile rearing environment and individual on dominant frequency and chirp rate indicated that individuals within families varied nonlinearly across the two rearing environments with respect to these two traits (Juv × Ind on *dfrq* and *crat* in Table 1, construction of F-tests in Table 3).

**Table 1.**
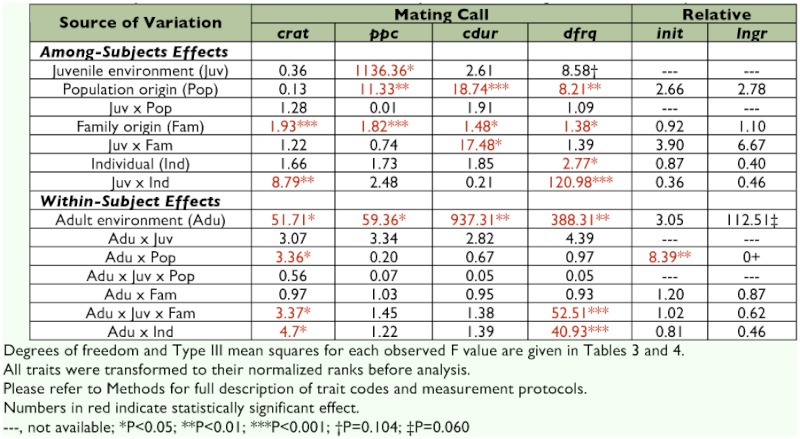
Summary of observed F values from statistical analyses of male mating call and female call preference.

**Table 2.**
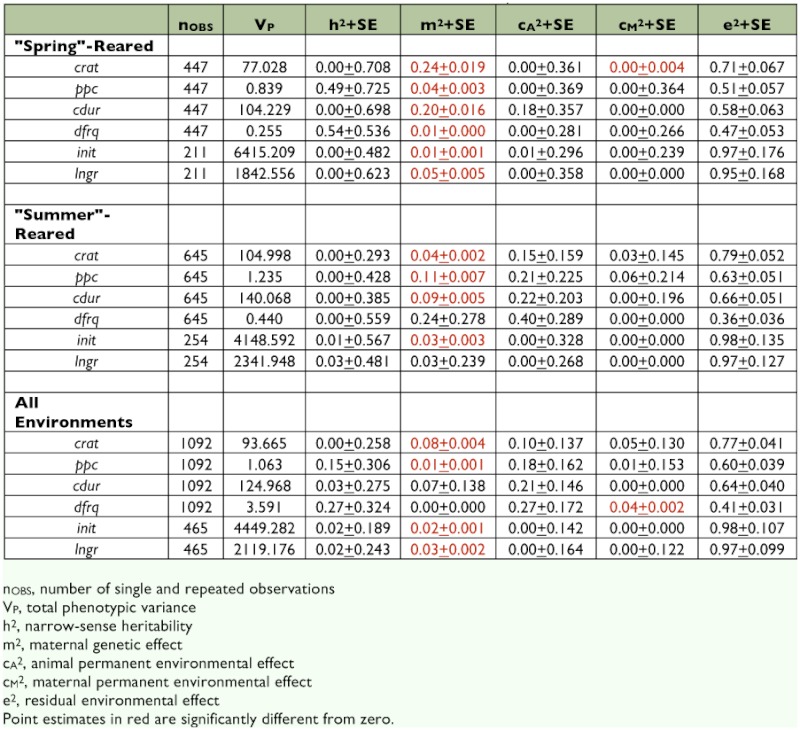
Restricted maximum likelihood estimates of model variance components.

Within each population, significant effects of family origin were detected on all four call components, though “family effects” on chirp duration indicated a two-way interaction with juvenile rearing environment (Juv × Fam on *cdur* in Table 1, construction of F-tests in Table 3). Quantitative-genetic analysis revealed that, aside from unexplained (residual) variance sources, maternal genetic factors accounted for an appreciable proportion of this effect, particularly in males reared under “spring-like” conditions (m^2^ column in Table 2).

None of the male calling song components showed significant heritability (h^2^). Point estimates reached as high as 54% of total phenotypic variation, as in the case for dominant frequency (*dfrq*) in spring-reared males, but were obscured by large standard errors (Table 2).

### Factors affecting female response to mating call

**Figure 3.**
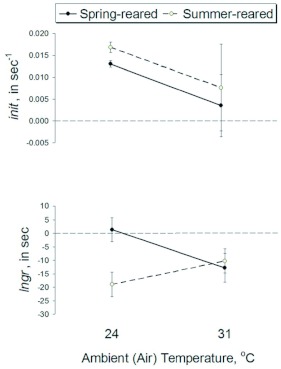
Environmental effects on call preference traits (mean ± 1 SE) of *Allonemobius socius* females. Relative preference of each female was scored once at each ambient temperature (*n* = 160 females): a positive score indicates female preference for the “summer-like” (or “hot”) male mating call, while a negative score indicates preference for the “spring-like” (or “cold”) male mating call. Trait codes are *init*: strength of initial association (i.e. inverse number of seconds spent walking toward a stimulus) made by females for “hot” versus “cold” male mating call and *lngr.* net directional preference in seconds of females for “hot” versus “cold” male mating call. See Methods for full description of trait codes and measurement protocols. High quality figures are available online.

Adult ambient temperature, as a main factor, had no consistent effect on female relative preference. The mostly positive *init* scores across the two ambient (adult) temperatures indicated that females initially associated with the “hot” call stimulus (Figure 3, upper panel). However, variation in a test subject's initial choice across the two adult temperature treatments was not statistically significant (Adu on *init* in Table 1, construction of F-tests in Table 4). The significant interaction between adult ambient temperature and population origin (Adu × Pop on *init* in Table 1, construction of F-tests in Table 4) indicated that females from the two sample populations approached the “hot” call stimulus in a nonlinear and inconsistent manner.

**Table 3.**
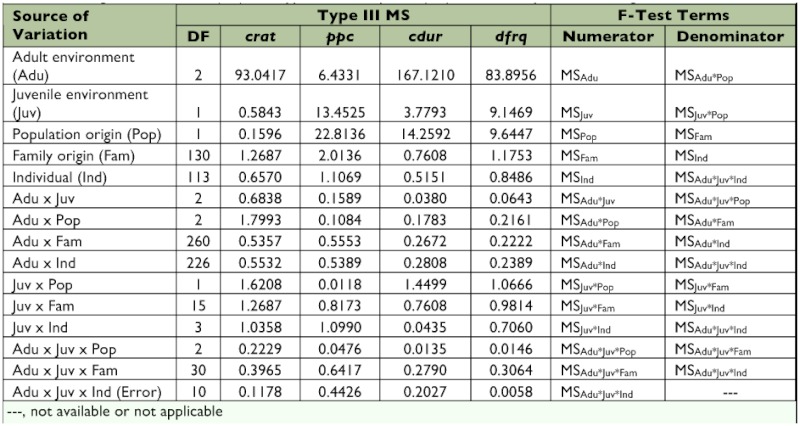
Degrees of freedom (DF) and Type III mean squares (MS) used to analyze male mating call.

Similarly, at both 24° C and 31° C, test subjects from either rearing environment tended to spend more time near the “cold” call stimulus than the alternative stimulus: The majority of *lngr* scores were negative, with such loitering behavior appearing more pronounced for summer-reared females (Figure 3, lower panel). However, the effect of ambient temperature on *lngr* scores was only marginally significant (Adu on *lngr* in Table 1, construction of F-tests in Table 4).

There was no effect of population or family origins on female relative preferences (Table 1, construction of F-tests in Table 4). Quantitative-genetic analyses on either *init* or *lngr* scores indicated no significant variance other than that from maternal genetic factors (m^2^ column for *init* and *lngr* in Table 2) and apart from large residual variation (e^2^ column in Table 2). However, these maternal genetic effects were small, accounting for no more than 5% of total phenotypic variance for either female trait, i.e. m^2^ < 0.05 for either *init* or *lngr* score in either environment (Table 2).

## Discussion

As the initial step in a complex reproductive repertory, female response towards male sexual signals provides the impetus for assortative mating and, hence, evolution of SMRS (Kirkpatrick 1982). While mate recognition in *A. socius* and other orthopterans likely involves other sensory modalities (Tregenza and Wedell 1997; Mullen et al. 2007), females generally recognize species-specific acoustic signals (Walker 1957; Olvido and Wagner 2004), though it remains less clear why between-species mating still occurs (Andersson 1994; Marler and Ryan 1997) or how exactly sexual choosiness and premating isolation might evolve within a species (Etges et al. 2007). More troubling, perhaps, is the finding from this study that different methods of quantifying female preference for call stimuli appear to yield diametrically different results (note the mostly positive *init* scores, suggesting relative preference for “hot” call stimulus across ambient temperatures, and the mostly negative *lngr* scores, suggesting relative preference for “cold” call stimulus across ambient temperatures (Figure 3) further illustrating the complexity of interpreting female responses to male mating call. Nonetheless, it is clear that environmental variation affects pre-mating behaviors in this species, and might explain in part the naturally occurring hybridization between *A. socius* and its more northern congener, *A. fasciatus.*

**Table 4.**
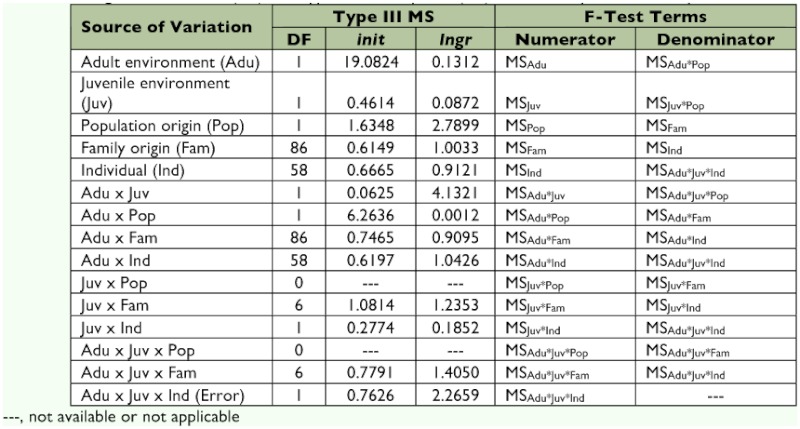
Degrees of freedom (DF) and Type III mean squares (MS) used to analyze female call preference.

### Environmental effects on male calling behavior

Though causing an apparent shift in mating call reaction norms, rearing environment, when compared with ambient temperature, had a small effect on the *A. socius* mating call. Across different taxa, variation in juvenile characteristics often translates to increased variation in the adult stage (Dingle 1996; Raff 1996; Walker 2000; Hebets 2003). And given the well-established correlation between ambient temperature and chirp rate of cricket mating calls (since Brooks 1882; Dolbear 1897; and until this study) (Figure 1), significant effects of juvenile environment were detected on only one of the four mating call components: pulses per chirp (*ppc* in Table 1) of summer-reared males appeared consistently lower than in their spring-reared full siblings (Figure 2). Failure to detect more widespread and pronounced juvenile environmental effects is most likely due to using a weak statistical test. A single degree of freedom in a repeated-measures ANOVA appears insufficient to detect relatively subtle adult phenotypic variation caused by variation in juvenile environment. It is also possible that any effect of juvenile environment may have “decayed” over the adult lifespan of the test subjects (Grace and Shaw 2004). Future studies should consider including more than two experimental levels of juvenile environment and a more precise accounting for adult age.

That juvenile environment did not fundamentally alter the shape of mating call reaction norms suggests physiological constraints on calling behavior, perhaps a reflection of expressed physiology-related genes that maintain the nature of a population- or even species-specific mating signal. Past studies on this and other gryllids have reported significant heritability of mating call components (Hedrick 1988; Webb and Roff 1992; Mousseau and Howard 1998), thus indicating a genetic basis for variation in such signaling traits. The current study, however, failed to detect heritable variation in all four mating call components (Table 2). Given the apparent absence of additive genetic variation in mating call components in this study, any attempt at estimating heritability of reaction norms for mating call seemed pointless. Future investigation into heritability of reaction norms will require far larger sample sizes and more extensive pedigrees than those reported here.

Family origin appears to be another major factor in male calling behavior, as it was significant for all four male mating call components (Table 1). The significant interaction of family origin and rearing environment on chirp duration (Juv × Fam on *cdur* in Table 1) suggested that, in terms of variation in chirp duration, males from different maternal lines respond non-uniformly to variation during their juvenile stages.

The quantitative genetic analysis revealed some “maternal genetic effects” (Table 2) on male mating call, i.e. heritable traits of mothers that shape offspring environment, which in turn affects male mating call. Given the absence of direct parental care in *A. socius,* any maternal genetic effect on mating call must be of a remote nature, e.g. maternal genes that affect the embryonic environment, including maternal gene products transferred into eggs (Yamashita 1996; Saino et al. 2005) and/or maternal choice of oviposition substrate (to the extent that oviposition behaviors are genetically determined). At present, however, it is not clear how the embryonic environment of *A. socius* might normally affect behavior manifested in its other lifecycle stages. Further investigation to quantify *A. socius* egg “quality” and maternal oviposition behavior certainly seems warranted.

### Environmentally mediated variation in female phonotaxis

Ambient temperature can affect *A. socius* female response to male mating call. Aside from their increased locomotory activity at higher temperatures, free-walking females tended to move toward the fast-chirp call stimulus much more quickly when observed at 24° C than at 31° C (Figure 3, top panel), though the difference in approach times between these two temperature treatments was not statistically significant (Table 1). And then, on any given trial, a female may abandon its first-chosen stimulus - a previously undocumented behavior - to spend significantly more time in the vicinity of the other, lower chirp-rate call (Figure 3, bottom panel).

Why *A. socius* females would show such a level of “acoustic promiscuity” is not clear. Searching for prospective mates, while apparently not physiologically costly to *A. socius* females, may prove costly in terms of increased predation risk (Walker and Masaki 1989). For example, most of the field collections prior to sorting and species identification contained various ground-dwelling spiders (presumably, *Lycosa* sp.) as by-catch. These large and fast-moving spiders seem capable of preying successfully on females that phonotactically locate prospective mates, though such predatory behavior was not observed when spiders were in accidentally prolonged confinement in the field-collected *A. socius* colonies.

Similarly, attraction to both fast-chirp and long-chirp stimuli in *A. socius* females may reflect a series of decisions that females make about a prospective mate. Perhaps females assess mate quality based on chirp rate (slow- versus faster-chirp mating calls) before further assessing mate quality based on chirp duration (short- versus long-chirp mating calls). Females may be attracted to multiple components of male mating call because different male traits provide independent and/or complementary information about fitness benefits (Wagner and Basolo 2007 and references therein). An earlier study of *A. socius* preference functions (Olvido and Wagner 2004) indicated that females generally associate with longer-chirp stimuli (i.e the “springlike” mating call stimulus in this study) and were less apt to associate with stimuli varying only in chirp rate. To the best of our knowledge, however, no previous study explored stimulus response in terms of the rate at which test subjects approach a given call stimulus, and thus cannot explain the lack of consistency between acoustic preference measured as approach behavior and acoustic preference measured as association behavior. In short, neither ambient temperature nor rearing environment explains why *A. socius* females approached the faster-chirp call stimulus sooner than the slow-chirp call and later preferred to associate with the longer-chirp call stimulus over the short-chirp alternative (Figure 3). Future studies should investigate more closely the relationship between female stimulus approach and association behaviors, as well as identify the relative importance of components, i.e. beyond chirp rate and chirp duration (Olvido and Wagner 2004) of *A. socius* mating call.

### On hybridization between *A. socius* and *A. fasciatus*

The persistence of natural hybrids produced from matings between *A. socius* and its more northern congener, *A. fasciatus,* continues to puzzle biologists. If intraspecific matings result in the highest possible offspring fitness (e.g. Groot et al. 2005), then why do *A. socius* females continue to mate with closely related heterospecifics? Furthermore, conspecific sperm precedence (Howard and Waring 1991) along with high population numbers, abundance of mobile individuals, many capable of long-distance flight in the wild (AO, personal observation), and widespread distribution (Marshall 2004) all indicate potential selection for intraspecific matings and selection against interspecific matings. So, why don't *A. socius-A. fasciatus* hybrids disappear from natural populations (Britch et al. 2001)?

One plausible explanation is that contemporary selection cannot yet suppress behaviors that lead to interspecific matings, at least in *A. socius.* An earlier study of individual preference functions established unequivocally the importance of the chirp structure of *A. socius* mating calls. Female *A. socius* responded positively to variation in *A. socius* mating calls and did not associate with the mating call typical of a sympatric trilling species, *Allonemobius tinnulus* (Olvido and Wagner 2004). The current study suggests that *A. socius* females normally approach conspecific mating calls, but will likely leave an *A. socius* male for a nearby *A. fasciatus* male, which produces a longer-chirp mating call (as estimated from Mousseau and Howard 1998). Thus, the current findings suggest that relatively promiscuous or confused *A. socius* females initiate interspecific matings and subsequently produce most of the naturally occurring hybrids, though similar promiscuity or confusion in *A. fasciatus* females expanding their range southward into *A. socius* populations cannot be ruled out. The northward range expansion of *A. socius* (Britch et al. 2001) seems more consistent with the former idea, however. Future studies should compare mating-call preference functions of *A. socius* females with those of *A. fasciatus* females.

## Abbreviations

SMRS: specific-mate recognition systems;
*crat*: chirp rate;
*ppc*: pulses per chirp;
*edur*: chirp duration;
*dfrq*: dominant frequency;
*init*: initial choice;
*lngr*: loitering behavior;
MTDFREML: multiple trait derivative-free restricted maximum likelihood
